# Concurrent chemoradiotherapy for T3–4 and N0–1 nasopharyngeal cancer: Asian multicenter trial of the Forum for Nuclear Cooperation in Asia

**DOI:** 10.1093/jrr/rrv046

**Published:** 2015-08-08

**Authors:** Tatsuya Ohno, Masaru Wakatsuki, Dang Huy Quoc Thinh, Ngo Thanh Tung, Dyah Erawati, Nana Supriana, C.R. Beena Devi, Shingo Kato, Kullathorn Thephamongkhol, Yaowalak Chansilpa, Miriam Joy C. Calaguas, Xu Xiaoting, Cao Jianping, Parvin Akhter Banu, Chul-Koo Cho, Kumiko Karasawa, Takashi Nakano, Hirohiko Tsujii

**Affiliations:** 1 Gunma University Heavy Ion Medical Center, Gunma University, Gunma, Japan; 2 Research Center for Charged Particle Therapy, National Institute of Radiological Sciences, 4-9-1 Anagawa, Inage-ku, Chiba 263-8555, Japan; 3 Department of Radiation Oncology No. 3, Ho Chi Minh City Cancer Center, Ho Chi Minh City, Vietnam; 4 Head and Neck Radiation Department, National Cancer Hospital, Hanoi, Vietnam; 5 Department of Radiotherapy, Dr Soetomo General Hospital, Surabaya, Indonesia; 6 Department of Radiotherapy, Dr Cipto Mangunkusumo General Hospital, Faculty of Medicine, University of Indonesia, Jakarta, Indonesia; 7 Department of Radiation Oncology, Sarawak General Hospital, Kuching, Malaysia; 8 Department of Radiation Oncology, Saitama Medical University International Medical Center, Saitama, Japan; 9 Department of Radiology, Faculty of Medicine, Siriraj Hospital, Mahidol University, Bangkok, Thailand; 10 Department of Radiation Oncology, St Luke's Medical Center, Quezon City, Philippines; 11 Department of Radiation Oncology, The First Affiliated Hospital of Soochow University, Suzhou, China; 12 Department of Radiation Oncology, Delta Hospitals Limited, Dhaka, Bangladesh; 13 Department of Radiation Oncology, Korea Institute of Radiological and Medical Sciences, Seoul, Korea; 14 Department of Radiation Oncology, Gunma University Graduate School of Medicine, Gunma, Japan

**Keywords:** nasopharyngeal cancer, chemoradiotherapy, cisplatin, 2-dimensional radiotherapy, developing country

## Abstract

The aim of this study was to evaluate the toxicity and efficacy of radiotherapy concurrent with weekly cisplatin for T3–4 and N0–1 nasopharyngeal cancer. Between 2005 and 2010, 70 patients with nasopharyngeal cancer (T3–4 N0–1 M0, World Health Organization Type 2–3) from Vietnam, Indonesia, Malaysia and Thailand were registered. Patients were treated with 2D radiotherapy concurrent with weekly cisplatin (30 mg/m^2^). Neither adjuvant nor induction chemotherapy was given. Ninety-three percent of the patients completed at least four cycles of weekly cisplatin during radiotherapy. The median total doses for the primary tumor and positive lymph nodes were 70 and 66 Gy, respectively. The median overall treatment time of concurrent chemoradiotherapy was 52 days. No treatment-related deaths occurred. Grade 3–4 acute toxicities of mucositis, nausea/vomiting and leukopenia were observed in 34%, 4% and 4% of patients, respectively. With a median follow-up time of 52 months for the 40 surviving patients, the 3-year local control, locoregional tumor control, distant metastasis–free survival and overall survival rates were 80%, 75%, 74% and 80%, respectively. In conclusion, the current results illustrate that our concurrent chemoradiotherapy regimen was feasible, but disease control remained insufficient. Further research is encouraged in order to improve clinical outcomes.

## INTRODUCTION

Nasopharyngeal cancer (NPC) is a special type of head and neck cancer usually found in South and Southeast Asian and North African populations. Radiotherapy (RT) is the mainstay treatment for NPC because of its surgically inaccessible anatomic location and radiosensitive character. Several randomized clinical trials and meta-analyses have demonstrated that concurrent chemoradiotherapy (CCRT) is the most efficacious approach for locoregionally advanced NPC [1–4].

It is important to identify patient subgroups likely to develop local failure or distant metastasis in order to establish individualized cancer treatment plans. Chua *et al*. reported 5-year local control (LC) and distant metastasis–free survival (DMFS) rates of 70% and 85%, respectively, for the T3–4 and N0–1 groups, 79% and 70%, respectively, for the T1–2 and N2–3 groups, and 76% and 60%, respectively, for the T3–4 and N2–3 groups. From these analyses of the failure pattern of NPC before the chemotherapy era, advanced local disease (T3–4) tended to be associated with local failure, whereas advanced nodal disease (N2–3) tended to be associated with distant metastasis [5, 6]. For the latter patient group with advanced nodal disease (any T and N2–3), we first conducted a prospective study of CCRT and adjuvant chemotherapy. We reported that the 3-year locoregional control (LRC), DMFS and overall survival (OS) rates were 89%, 74% and 66%, respectively, for 121 patients registered from Vietnam, Malaysia, Indonesia, Thailand, the Philippines, China and Bangladesh [7]. In contrast, for patients with advanced local disease (T3–4 and N0–1), it is unknown whether induction or adjuvant chemotherapy is necessary. Therefore, as a second clinical study, we initiated a prospective study of CCRT without induction or adjuvant chemotherapy in this patient group. The purpose of this study was to evaluate the efficacy and toxicities of this regimen, especially in Southeast Asian countries, including Vietnam, Indonesia, Malaysia and Thailand, where NPC is endemic.

## MATERIALS AND METHODS

This study was conducted within the Forum for Nuclear Cooperation in Asia (FNCA), a framework of regional cooperation among Asian countries supported by the Japanese government with the aim of peaceful and safe application of nuclear science and technology. The medical project of the FNCA was launched in 1993, aiming to standardize RT and CCRT for common cancers in Asia such as cervical cancer and NPC, with the participation of 11 Asian countries: Bangladesh, China, Indonesia, Japan, Kazakhstan, Korea, Malaysia, Mongolia, the Philippines, Thailand and Vietnam [8].

### Patient eligibility

A multi-institutional, prospective, single-arm study was designed. Patients fulfilling all the following criteria were eligible for this study: histologically confirmed World Health Organization (WHO) Type 2 or 3 carcinoma of the nasopharynx, Stage III or IVA disease with T3–4 and N0–1 classification (UICC-TNM, 6th edition), age between 20 and 70 years, performance status (PS) 0–2, adequate bone marrow, hepatic, and renal function (WBC count ≥ 3000/mm^3^, Hb ≥ 10 g/dl, platelets ≥ 100 000/mm^3^, total bilirubin ≤ 1.5 mg/dl, AST/ALT ≤ 2× upper limit of normal, serum creatinine ≤ 1.5 mg/dl). The exclusion criteria were WHO Type 1 carcinoma of the nasopharynx, severe concomitant illness such as uncontrolled cardiovascular disease, uncontrolled diabetes mellitus, active peptide ulcer, severe infection, severe psychological illness, an active double cancer, prior RT or chemotherapy, and pregnancy or lactation. Written informed consent was obtained from all patients. All patients underwent nasopharyngoscopy and biopsy to obtain specimens for pathological diagnosis. Pretreatment evaluations included physical examination of the head and neck, computed tomography (CT), chest radiography, a complete blood cell count with differential counts, and a biochemistry profile. Because of differences in the availability of medical resources among the participating institutes, abdominal ultrasonography and bone scans were used optionally, but their use was recommended if possible.

### Radiotherapy

Patients were treated using a 6- or 10-MV linear accelerator or a telecobalt unit via a conventional 2D-RT technique. The use of CT, magnetic resonance imaging (MRI), and nasopharyngoscopy is recommended to define the gross tumor. The superior margin of the initial radiation field ranged 2 cm beyond the visible tumor on CT and included the entire base of the skull and the sphenoid sinus. Posteriorly, the field extended at least 1.5 cm beyond palpable nodes. Anteriorly, the field included the posterior ethmoidal sinus, the posterior one-third of the maxillary antrum, or at least 1.5 cm beyond the visible tumor. Patients received conventional fractionated RT (1.8–2 Gy per fraction, five daily fractions per week). Patients were treated in a supine position, usually with bilateral parallel opposing fields to the primary tumor and upper neck and a single anterior field to the lower neck with a central shield. After 40–45 Gy of radiation was delivered, the primary tumor was boosted using bilaterally opposed reduced portals. The bulky nodal area was irradiated with posterio–anterio parallel opposing ports for the neck region or an electron beam with appropriate energy. The total planned dose was 66–70 Gy for T3 lesions, 66–75 Gy for T4 lesions, and 60–70 Gy for the positive neck region. RT was suspended if a patient developed Grade 4 hematological toxicities, Grade 4 radiation mucositis of the oral cavity or pharynx, Grade 4 radiation dermatitis, ≥ Grade 3 non-hematological toxicities (e.g. nausea, vomiting) excluding mucositis and/or dermatitis, or PS 3–4. RT was resumed when the hematological and non-hematological toxicities recovered to Grade 2.

### Chemotherapy

Cisplatin at a dose of 30 mg/m^2^ was administered weekly starting from Week 1 for six consecutive weeks during the course of RT. Patients were hydrated with more than 1500 ml of normal saline per session. As antiemetics, 5-HT3 receptor antagonists and dexamethasone were given with the chemotherapy. The administration of cisplatin with RT was interrupted when patients developed a WBC count < 3000/mm^3^, a platelet count < 75 000/mm^3^, fever > 38.0°C, PS 3–4, ≥Grade 3 non-hematological toxicities (e.g. emesis, loss of appetite, fatigue), or serum creatinine > 1.5 mg/dl.

### Assessment and follow-up

While patients were undergoing CCRT, toxicity and tumor response were evaluated weekly. The Common Terminology Criteria for Adverse Events v3.0 was used to evaluate toxicities. After treatment, follow-up examinations were conducted at least every 3 months for the initial 3 years and then every 3–6 months for the subsequent 2 years. Disease status and toxicities were assessed by physical examination, appropriate laboratory tests, and chest radiography. Imaging modalities such as ultrasonography, CT or MRI were used if necessary. The LC, LRC, DMFS and OS rates were estimated using the Kaplan–Meier method. An annual meeting was held to review the patients' eligibility, treatment technique employed in the study, toxicities and follow-up status in each center.

### Statistical analysis

The primary endpoint of the study was the 3-year OS rate. The secondary endpoints included the 3-year LC rate, 3-year LRC rate, 3-year DMFS rate, acute toxicities and late toxicities. Based on the retrospective analysis of clinical data among the participating institutes of the FNCA project, the 3-year OS rate with 2D-RT alone for patients with locoregionally advanced NPC was 60%. The sample size evaluated in this study, which was calculated using the 3-year OS rate, was determined to be 100 patients. We chose a rate of 80% as a desirable target level and a rate of 60% as undesirable. Our design had a power in excess of 80% and a Type I error of less than 5%. Considering a decrease in power (e.g. loss to follow-up and entry of ineligible cases), this trial was designed to enroll 100 patients. The actual LC, LRC, DMFS and OS rates were calculated using the Kaplan–Meier method.

## RESULTS

### Patient characteristics

Between April 2005 and May 2010, 70 patients were enrolled from Ho Chi Minh City Cancer Center (Vietnam), National Cancer Hospital (Vietnam), Sarawak General Hospital (Malaysia) and Siriraj Hospital (Thailand). However, no new patient has been enrolled since May 2010, mainly because of competing clinical trials for NPC at each center and the relatively lower incidence of the disease. Following a discussion at the FNCA Workshop on Radiation Oncology in November 2013, enrollment in the current clinical trial was discontinued prematurely. Analysis was performed on all data entered at the FNCA data center as of 30 October 2013. The median follow-up period was 49 months for all patients, versus 52 months for the 40 surviving patients. The patient characteristics are listed in Table 1. For the pretreatment evaluation, CT scans for head and neck, bone scans and ultrasonography of the upper abdomen were performed for 59 (84%) and 67 patients (96%), respectively.

**Table 1.** Patient characteristics (*n* = 70)

Age (years)		
Median (range)	49 (27–65)	
	*n*	(%)
Gender		
Male	55	(79)
Female	15	(21)
Performance status		
0	21	(30)
1	35	(50)
2	14	(20)
T classification		
T3	41	(59)
T4	29	(41)
N classification		
N0	15	(21)
N1	55	(79)
Clinical stage		
Stage III	41	(59)
Stage IVA	29	(41)
WHO classification		
Type 2	8	(11)
Type 3	62	(89)

### Treatment and compliance

A total of 39 patients (56%) were treated with the linear accelerator, and 31 patients (44%) were treated with a telecobalt unit. The median total doses for the primary tumor and positive lymph nodes were 70 and 66 Gy, respectively. The median overall treatment time of CCRT was 52 days. Of the 70 patients, 15 (21%) required interruption of RT. Five patients (10%) required interruption for more than 14 days, with the median duration of interruption being 7 days. The reasons for interruption of RT were acute non-hematological toxicities such as mucositis, pain and dermatitis in five patients, hematological and non-hematological toxicities in three patients, machine malfunction in three patients, and others (e.g. public holiday) in four patients. Of the 70 patients, 52 (74%), 10 (14%), 4 (6%) and 4 (6%) patients received 6, 5, 4 and 1–3 cycles of concurrent chemotherapy, respectively. The reasons for incomplete concurrent chemotherapy were treatment-related toxicities in 17 patients, patient refusal in 4 patients, and poor general condition in 1 patient.

### Toxicities and efficacy

The hematological and non-hematological acute toxicities are listed in Table 2 and 3. One patient developed Grade 4 nausea/vomiting and anemia. The incidences of ≥Grade 3 mucositis, nausea/vomiting and leucopenia were 25.7%, 1.4% and 4.3%, respectively. The late toxicities are listed in Table 4. The incidences of ≥Grade 3 skin toxicity and dry mouth were 13% and 63%, respectively.

**Table 2.** Hematological acute toxicities

Toxicity	Grade (CTCAE ver. 4)				
	0	1	2	3	4
Leukopenia	25	19	23	3	0
Neutropenia	34	22	9	5	0
Anemia	35	26	7	1	1
Thrombocytopenia	47	19	4	0	0

**Table 3.** Non-hematological acute toxicities

Toxicity	Grade (RTOG/EORTC)				
	0	1	2	3	4
Dermatitis	4	32	27	7	0
Mucositis	2	25	25	18	0
Pain	9	35	21	5	0
Dry mouth	3	31	34	2	0
Nausea/Vomiting	21	37	11	0	1
Weight loss	24	21	22	3	0
Fatigue	26	18	24	1	1

**Table 4.** Late toxicities

Toxicity	Grade (RTOG/EORTC)				
	0	1	2	3	4
Subcutaneous	8	33	29	0	0
Mucosa	4	55	11	0	0
Skin	6	31	24	9	0
Dry mouth	0	12	14	44	0

**Fig. 1.**
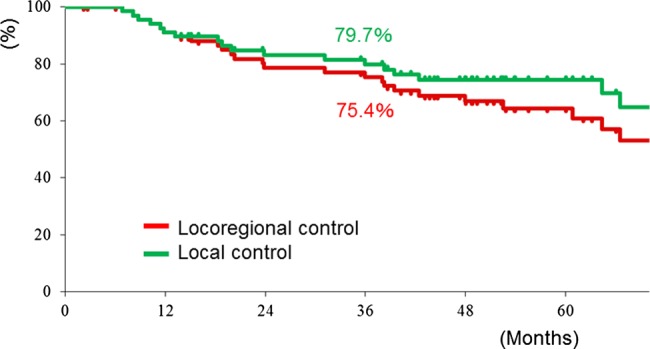
Local control (LC) and locoregional control (LRC) rates for all 70 patients.

**Fig. 2.**
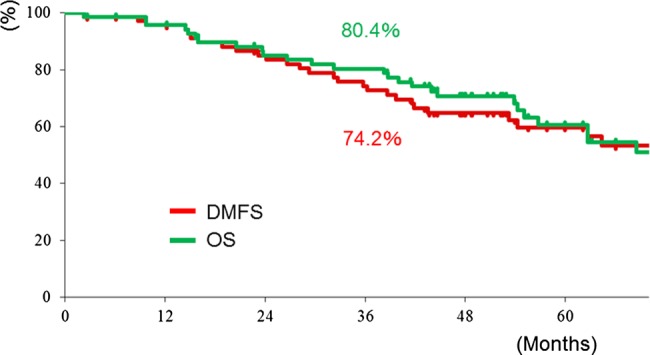
Overall survival (OS) and distant metastasis-free survival (DMFS) rates for all 70 patients.

The first sites of failure were locoregional sites in 21 patients, distant sites in 9 patients, and both locoregional and distant sites in 4 patients. Among the 25 patients with locoregional failure, the first failure sites were the primary lesion, lymph nodes and both in 14, 8 and 3 patients, respectively. The 3-year LC and LRC rates for all 70 patients were 80% and 75%, respectively (Fig. 1). The 3-year DMFS and OS rates for all 70 patients were 74% and 80%, respectively (Fig. 2).

## DISCUSSION

In the present study, CCRT without induction or adjuvant chemotherapy was administered to patients with NPC (T3–4, N0–1) in Southeast Asian centers. The 3-year OS rate of 80% was relatively better than that of our historical control (60%; RT alone), but lower than recent published data in leading Asian facilities (85–90%; intensity-modulated radiation therapy [IMRT] with or without chemotherapy) [9]. Several points need to be considered when interpreting the clinical outcomes in this study. First, the target disease focused on a select disease category (T3–4 and N0–1) that is an uncommon form of NPC. Second, these clinical data were obtained mainly from Vietnam, Indonesia and Malaysia, where the English literature on CCRT for NPC is extremely limited, despite the high incidence of NPC [10]. Third, in the regions studied, most patients present with advanced disease, and the number of patients requiring RT generally exceeds the limited medical resources.

The present study suggested that T3–4 primary tumors were not sufficiently controlled by concurrent weekly cisplatin and 2D-RT. One possible reason is that compared with reports on IMRT, the 2D-RT technique used in our study contributed to inferior tumor control because IMRT could improve target coverage and normal tissue sparing. Su *et al*. reported 5-year LRC, DMFS and OS rates for patients with T3–4 and N0–1 NPC who were treated with IMRT with or without chemotherapy of 87%, 84% and 80%, respectively, which were relatively better than our outcomes [11]. Recently, other studies on IMRT also reported LRC rates of 81–96% for T3–4 tumors [12–14]. Additionally, a Phase III study illustrated the superiority of IMRT over 2D-RT in terms of LRC, OS and late toxicities [15]. Unfortunately, IMRT was not available in all participating centers during the study period, and the primary curative treatment mainly relied on 2D-RT.

The other possible reason for unfavorable LC was that the dose for the primary tumor (median dose, 70 Gy) in our study was insufficient. Teo *et al*. investigated the effect of dose escalation above a conventional tumor dose level of 66 Gy when the basic RT course was delivered by 2D-RT [16]. For T3–4 tumors, the administration of boost radiation, mainly with 2D-RT, (the median dose, 10 Gy) after 66 Gy significantly improved local tumor control. Increasing the dose for the primary tumor site appears promising; however, dose escalation with 2D-RT also increases the risk of late toxicities such as chronic radiation necrosis, hearing loss, dysphagia and temporal lobe necrosis. Kwong *et al*. reported that an IMRT dose of 76 Gy for T3–4 tumors in combination with cisplatin-based chemotherapy was associated with a 2-year LC rate of 96%, but 84% of the patients developed ≥Grade 3 ototoxicity [12]. Several attempts have been made regarding dose escalation using 3D conformal techniques, stereotactic RT, and IMRT [9]; however, there is no standardized RT technique including the dose and fractionation schedule.

Our study indicated that CCRT alone for patients with T3–4 and N0–1 tumors was not adequate to control distant metastasis. In particular, because of the insufficient LRC, our data included secondary metastasis after locoregional failure. Thus, efforts should be made to minimize locoregional recurrence. In contrast, an increased possibility of distant failure was noted when the tumor invaded the bone marrow of the skull base or parapharyngeal venous plexus [17]. Su *et al*. observed similar DMFS rates between advanced local disease (T3–4 and N0–1) and advanced nodal disease (T1–2 and N2–3) groups [11]. To reduce systemic failure and improve survival, the combination of CCRT with induction or adjuvant chemotherapy has been also investigated. A recent Phase III randomized clinical trial comparing CCRT alone with CCRT and adjuvant chemotherapy in patients with locoregionally advanced NPC indicated that adjuvant chemotherapy consisting of cisplatin and 5-FU added little survival benefit to CCRT [13]. Additionally, two recent meta-analyses demonstrated that adjuvant chemotherapy after CCRT did not confer a statistically significant survival improvement compared with RT alone or CCRT [4, 18]. In contrast, the additional benefit of induction chemotherapy to CCRT for locally advanced NPC remains unclear. Fountzilas *et al*. reported a randomized Phase II study demonstrating that three cycles of induction chemotherapy followed by CCRT did not provide a significant survival benefit compared with CCRT alone [19]. Ongoing Phase III randomized trials will further explain the role of induction chemotherapy in combination with CCRT.

Our treatment regimen was feasible with manageable toxicities. When we consider an increase in the treatment intensity, however, care should be exercised because dose escalation or the addition of chemotherapy to CCRT will increase the risk of treatment-related toxicities. In developing countries in particular, the general conditions of the patients are compromised, and they could become further compounded by the lack of adequate supportive therapy for managing treatment-related toxicities [20, 21].

A long diagnosis-to-treatment with RT interval and a prolonged overall RT treatment time for head and neck cancers have been considered unfavorable prognostic factors. Stoker *et al*. reported that the median diagnosis-to-treatment interval for RT for NPC in one Indonesian center was 106 days because of the lack of a sufficient RT unit, and the median overall treatment time was extended by 10–12 days because of RT machine malfunction (36%), patients' poor condition (21%), and public holidays (14%) [22]. A similar survey was also reported from Taiwan [23]. The mean diagnosis-to-treatment interval for RT for NPC and the overall treatment time were 13 and 68 days (∼12 days of excess), respectively. Thus, major differences exist in the diagnosis-to-treatment interval for RT, which may have contributed to the divergent clinical outcomes. In our study, the median OTT was 52 days, in which machine malfunction and holidays were included as causes of RT interruption, whereas the diagnosis-to-treatment interval for RT was not measured. As the participating centers in the present study also had similar diagnosis-to-treatment intervals for RT, cancer might progress during the waiting time, resulting in more advanced disease.

In summary, 2D-RT concurrent with weekly cisplatin in patients with T3–4 and N0–1 NPC was feasible with manageable toxicities in the participating centers. However, the treatment regimen appeared insufficient for controlling both locoregional and distant disease. Further research is encouraged in order to improve clinical outcomes, especially focusing on the combination of high-precision RT modalities such as IMRT with systemic chemotherapy. Whereas, our clinical trial was divided between advanced nodal disease (N2–3) and advanced local disease (T3–4 and N0–1) groups, this protocol was conducted for patients with advanced local disease (T3–4 and N0–1). On the other hand, we first conducted a prospective study of CCRT and adjuvant chemotherapy for patients with advanced nodal disease (N2–3). In addition, we are now conducting a new clinical trial for patients with advanced nodal disease (N2–3) using induction chemotherapy followed by concurrent chemoradiotherapy.

## FUNDING

This work was supported by the project of The Forum Nuclear Cooperation in Asia (FNCA), the Ministry of Education, Culture, Sports, Science and Technology (MEXT) of Japan, and the research project of the National Institute of Radiological Sciences, Japan. Funding to pay the Open Access publication charges for this article was provided by the research project of the National Institute of Radiological Sciences, Japan.
